# Acute Kidney Injury in Patients Admitted to the Intensive Care Unit: A Case Report

**DOI:** 10.7759/cureus.40380

**Published:** 2023-06-13

**Authors:** Ayman Mohamed, Michael Peniston, Rabia Mahmood

**Affiliations:** 1 Internal Medicine, Ascension St. John Hospital, Detroit, USA; 2 Internal medicine, Ascension St. John Hospital, Michigan, USA

**Keywords:** ercp, critical care, : acute kidney injury, cholemic nephropathy, medical intensive care unit (micu)

## Abstract

Patients admitted to the intensive care unit are prone to various complications, one of which is acute kidney injury (AKI). The etiology of acute kidney injury can be multifactorial. Among the various causes, sepsis remains the most prevalent. Cholemic nephropathy (CN) is a rare cause of AKI. Patients with CN usually present with elevated total bilirubin levels of greater than 20 mg/dl. However, CN has been reported in patients with total bilirubin levels of less than 20 mg/dL. These patients were found to have chronic elevations of bilirubin due to chronic liver disease rather than an acute rise in bilirubin levels. In this case series, we present two cases of patients with chronic liver disease who were admitted to the intensive care unit and were found to have AKI with elevated total bilirubin levels over 15 mg/dl.

## Introduction

Acute kidney injury (AKI) is a frequent complication among patients admitted to intensive care units (ICUs), with reported incidences ranging between 30% and 60% [1]. Among the various causes of AKI, sepsis is recognized as the most common etiology, with urinary tract infections being the primary source [2]. In a retrospective study examining 500 patients with AKI in the ICU setting, sepsis was identified as the cause of AKI in 38.6% of patients. In addition to sepsis, other potential causes of AKI in critically ill patients include cardiac and hepatic etiologies, drug-induced kidney injury, as well as cholemic nephropathy (CN). This case report provides a clinical report on two patients who were admitted to the intensive care unit (ICU) due to sepsis and were subsequently diagnosed with acute kidney injury (AKI). Notably, both patients demonstrated elevated total bilirubin levels, prompting suspicion of a plausible link between chronically elevated bilirubin levels and the development of cholemic nephropathy.

## Case presentation

Case 1

A 64-year-old woman with a recent diagnosis of pancreatic adenocarcinoma, hypertension, hypothyroidism, and asthma presented to the hospital with complaints of fatigue. Her symptoms had gradually worsened over the past two months, accompanied by frequent diarrhea, with two episodes of loose stools per day, and decreased urine output. She had received one cycle of chemotherapy with Leucovorin, Fluorouracil, Irinotecan, and Oxaliplatin previously and was scheduled for a second cycle, which was deferred due to her current symptoms.

Two months prior to the patient's current presentation, a computed tomography (CT) scan of the abdomen and pelvis had revealed the presence of a 3.4 x 2.1 x 3.9 cm mass originating from the head of the pancreas, which caused obstruction of the common bile duct and resulted in intrahepatic bile duct dilation. Given the patient's symptoms, an ERCP was performed around the same time, which revealed distal common bile duct stricture with dilation upstream. The patient underwent sphincterotomy with biliary duct stent placement during that procedure. Subsequent biopsy of the mass demonstrated rare atypical cells consistent with pancreatic adenocarcinoma.

**Figure 1:**
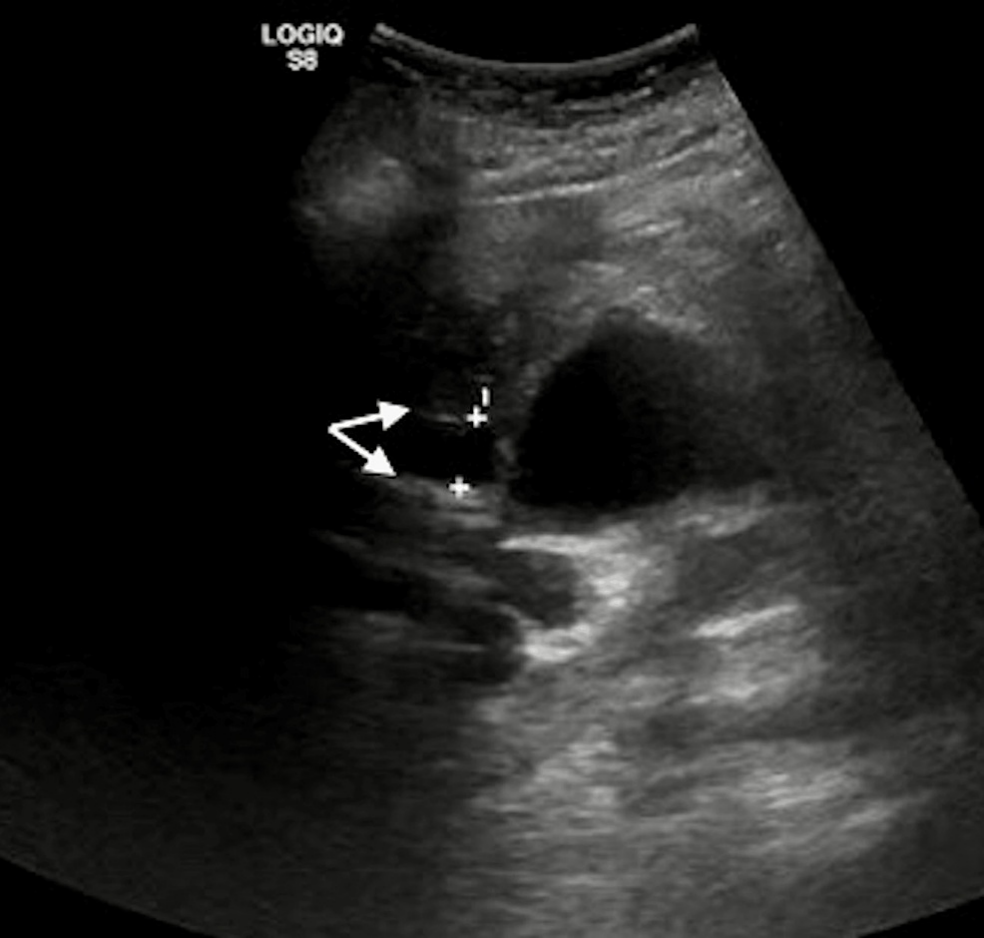
An ultrasound of the abdomen showing dilation of the common bile duct measuring approximately 1.5 cm in diameter

Upon initial presentation, the patient was hypotensive, tachycardia, and tachypneic. Physical examination was remarkable for mild abdominal tenderness in the right upper quadrant and mild abdominal distention. Table 1 shows the lab values on admission. A chest x-ray showed no discernible abnormalities. An ultrasound of the abdomen revealed intrahepatic bile duct dilation, involving the common bile duct which measured approximately 1.5 cm in diameter (Figure 1).

**Table 1:** Lab values on admission

Variable	Value on admission	Reference range
Sodium mmol/L	127	135-145
Bicarbonate mmol/L	13	23-34
Anion gap mmol/L	24	4-14
Total bilirubin mg/dl	16.9	0-1.5
Direct bilirubin mg/dl	9.4	0-0.4
Alkaline phosphatase IUnits/L	360	20-130
Aspartate aminotransferase IUnits/L	137	0-45
Alanine aminotransferase Unit/L	67	0-45
Total Protein gm/dl	5.1	6.2-8.1
Albumin gm/dl	2.7	3.5-5
Lactic acid mmol/L	3	0.5-2
Lipase IUnits/L	11	13-60
Creatinine	Unable to determine on admission	0.7-1.5

Notably, a previously placed stent was observed in the duodenum but not in the common bile duct. Following this, an endoscopic retrograde cholangiopancreatography (ERCP) was performed, which revealed an irregular stricture in the distal common bile duct along with upstream ductal dilation of around 1.5 cm. Pus was drained from the biliary tree, and an attempt to place a biliary stent via ERCP was unsuccessful. As an alternative approach, an external biliary drain catheter was subsequently placed via interventional radiology on the third day of the patient's admission. Subsequent culture of the drained pus from the biliary tree showed growth of *Serratia marcescens*, *Enterococcus fecalis*, and *Enterobacter cloacae*. Blood cultures also yielded positive results for *Enterobacter cloacae *and* Klebsiella oxytoca*.

The patient was transferred to the medical intensive care unit and initiated on norepinephrine, epinephrine, and vasopressin, requiring three vasopressors for blood pressure support. Broad-spectrum antibiotics, including cefepime, vancomycin which was adjusted due to worsening renal function, and metronidazole, were initiated. However, given the persistence of fever and the deterioration in renal function, a decision was made to switch to intravenous meropenem. The patient's urinary output exhibited a decline from 465 cc on the first day to 105 cc on the second day. Following the administration of a single intravenous dose of furosemide at 40 mg, an improvement was observed in the urine output, which reached 563 cc on the third day. Subsequently, a furosemide infusion was initiated, resulting in a further enhancement of urinary output. A retroperitoneal ultrasound examination was performed, which did not reveal the presence of any masses but indicated the presence of bilateral hydronephrosis. As the patient's hemodynamic status gradually improved, the vasopressors were slowly tapered to one pressor. However, on the ninth day of admission, the patient's bilirubin levels increased, as shown in Table 2, documenting the trends in bilirubin and creatinine levels.

**Table 2:** Total bilirubin and creatinine trends during admission

Day	Creatinine (0.7-1.5)	BUN (8-20)	Total bilirubin (0-1.5)
Third	2.59	48	15.6
Fourth	2.96	47	11.9
Fifth	3.52	53	9.2
Sixth	3.83	62	7.7
7th	3.84	67	8.3
8th	4	72	7.2
9th	4.05	85	7.8
10th	3.85	95	8
11th	4.15	108	9.3
12th	4.35	106	11.1
13th	4.47	123	11.2
14th	4.85	132	12

**Figure 2:**
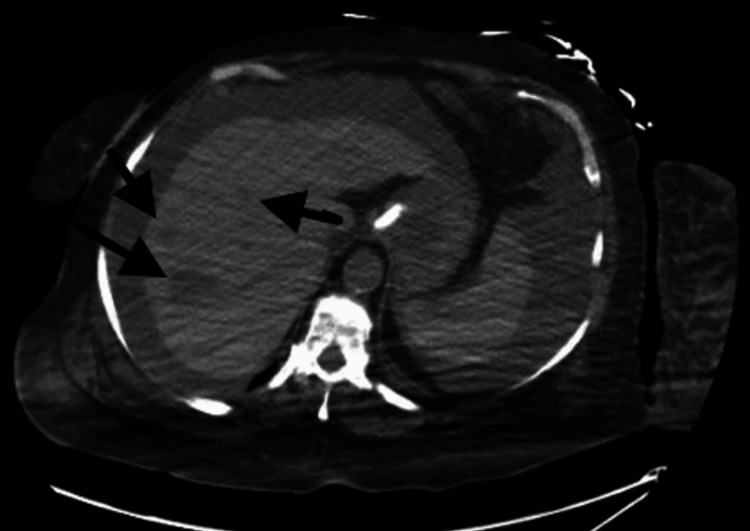
Computed tomography of the abdomen without contrast showing multiple right hepatic lesions.

A right upper quadrant ultrasound was performed, revealing no intrahepatic bile enlargement but showing right-sided hydronephrosis. A CT scan of the abdomen without contrast showed multiple right hepatic lesions, raising suspicion for a metastatic process (Figure 2), in addition to the right-sided hydronephrosis and hydroureter. Given the advancing malignancy and worsening sepsis, and after having further discussions with the family, comfort care measures were pursued.

Case 2

A 67-year-old male patient with a medical history of chronic obstructive pulmonary disease (COPD), prior intravenous drug use, chronic hepatitis C, tobacco use, and a recent diagnosis of metastatic small cell lung carcinoma presented with altered mental status. His last recorded normal cognitive state was observed approximately 24 hours prior to his arrival at the hospital. Upon admission, the patient was unable to provide any substantial medical history due to his compromised mental state. Consequently, contact was made with the patient's family, who reported that he had experienced an acute onset of confusion on the day preceding his hospital admission. Notably, he was recently diagnosed with stage IV small cell lung cancer and had a scheduled treatment plan for palliative chemotherapy, which had not yet commenced at the time of the patient's presentation.

**Figure 3:**
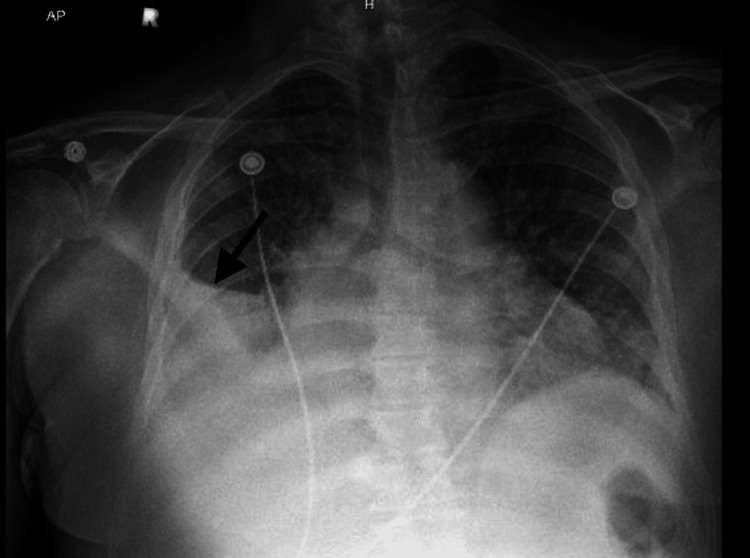
Chest X-ray showing right pleural effusion.

**Figure 4:**
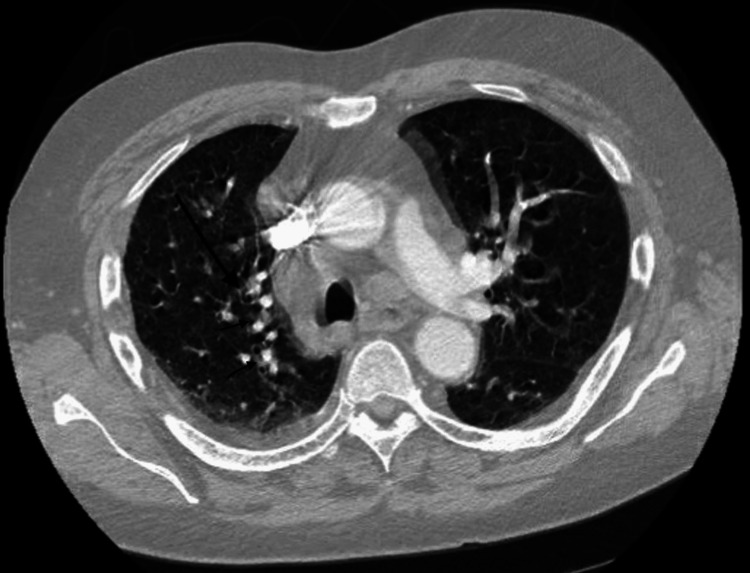
Computed tomography (CT) angiography showing multiple masses within the right perihilar lung and mediastinum, consistent with metastatic disease

On initial presentation, the patient was hypothermic, and tachycardic with a heart rate in the 130s, saturating 100% on a 3L nasal cannula, and a blood pressure of 108/61. Blood cultures were obtained and broad-spectrum antibiotics were started. The patient was noted to have pitting edema present up to the level of his abdomen. Table 3 shows the patient’s pertinent lab values on presentation to the hospital. Chest x-ray showed evidence of interstitial edema with an increasing right pleural effusion (Figure 3). Computed tomography (CT) angiography showed no evidence of pulmonary embolism but was significant for multiple masses within the right perihilar lung and mediastinum, consistent with metastatic disease (Figure 4). No acute findings were found on the CT head without contrast.

**Table 3:** Lab values on admission

Variable	Value on admission	Reference range
Sodium mmol/L	139	135-145
Bicarbonate mmol/L	21	23-34
Anion gap mmol/L	13	4-14
Total bilirubin mg/dl	18.2	0-15
Direct bilirubin mg/dl	14	0-4
Alkaline phosphatase IUnits/L	296	20-130
Aspartate aminotransferase IUnits/L	228	0-45
Alanine aminotransferase Unit/L	62	0-45
Total Protein gm/dl	Unable to be determined	6.2-8.1
Albumin gm/dl	3	3.5-5
Lactic acid mmol/L	3.3	0.5-2
Creatinine mg/dl	1.92	0.7-1.5
BUN mg/dl	50	8-20
Urine Sodium mmol/L	< 20	-
Urine Creatinine mg/dl	124.3	> 20

**Figure 5:**
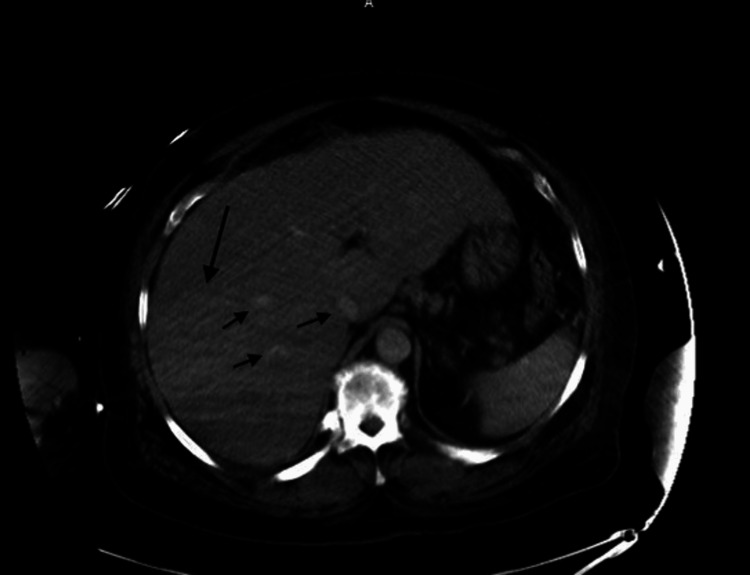
CT of the abdomen and pelvis showing hepatomegaly and heterogeneous parenchyma concerning for cirrhosis or metastatic lesions

A CT of the abdomen and pelvis revealed the presence of hepatomegaly and heterogeneous parenchyma, which raised concerns regarding the potential existence of cirrhosis or metastatic lesions (Figure 5). Consequently, the patient underwent a comprehensive evaluation conducted by the gastroenterology service to address the significant hyperbilirubinemia. In light of the clinical assessment, it was determined that the hyperbilirubinemia was likely a secondary manifestation of the patient's extensive metastatic disease, thus prompting the recommendation of supportive care. Notably, based on the imaging findings, there was no compelling indication to pursue endoscopic retrograde cholangiopancreatography (ERCP) due to the absence of observed ductal obstruction.

The patient's hospital course was marked by significant challenges, including increased work of breathing and reduced tolerance for BiPAP (bilevel positive airway pressure) therapy. As a result, the patient's deteriorating condition necessitated transfer to the Intensive Care Unit (ICU) following intubation to ensure airway protection, along with the initiation of norepinephrine and vasopressin for hemodynamic support. Subsequently, the patient presented with melena and experienced a substantial decrease in hemoglobin levels, necessitating the administration of a packed red blood cell transfusion. Furthermore, there was a notable decline in the patient's urine output from 415 cc on the first day to less than 100 cc on the second day.

In light of the patient's unfavorable prognosis, a treatment plan centered around supportive care was devised. Ultimately, after engaging in comprehensive discussions with family members, it was collectively determined that the implementation of comfort care measures was most appropriate.

## Discussion

Patients requiring admission to the intensive care unit (ICU) often present with multiple comorbidities, some of which can impact kidney function and increase the risk of developing acute kidney injury (AKI). Among various etiologies contributing to AKI, sepsis remains the predominant cause [2]. Within the context of sepsis-induced AKI, urinary tract infections (UTIs) frequently serve as the primary source. Other causes of AKI related to liver dysfunction include hepatorenal syndrome, which is a diagnosis of exclusion made after other possible etiologies are ruled out. The patients described in this case report exhibited several risk factors, including sepsis, predisposing them to the development of AKI. Nonetheless, these patients also presented with supplementary factors that may have potentially played a role in the development of acute kidney injury (AKI). This is evident through the observation of elevated bilirubin levels and the potential occurrence of cholemic nephropathy.

CN, also known as bile cast nephropathy (BCN), is a pathological condition characterized by the acute impairment of renal function in patients with hepatic dysfunction [3]. This disorder is commonly associated with marked hyperbilirubinemia and the development of tubular injury due to bile cast formation [3, 4]. The formation of bile acid casts results from elevated bilirubin levels, which stimulate the increased excretion of bile acids. This process is exacerbated by various factors, including metabolic acidosis [4]. Numerous liver pathologies, including acute cholangitis and obstructive jaundice, have been linked with the onset of AKI. In a retrospective study involving 1438 patients diagnosed with acute cholangitis, the primary objective was to investigate the possible association between acute cholangitis and the occurrence of acute kidney injury (AKI), as well as its implications for prognosis [5]. The findings of the study revealed that a notable proportion of patients (18.4%) diagnosed with acute cholangitis subsequently progressed to develop AKI [3].

The two cases presented in this case report have demonstrated sepsis as the probable etiology of acute kidney injury (AKI). However, the patients' total bilirubin levels remained elevated and bile cast formation was observed on microscopy. Bile cast nephropathy is frequently observed in patients with markedly elevated bilirubin levels, specifically above 20 mg/dL [6]. However, a study that was conducted to assess the presence of bile casts in 114 autopsies of patients with cirrhosis found that bile casts may develop in patients with chronic liver disease, even when their bilirubin levels are less than 20 mg/dl. Moreover, the study suggests that the formation of bile casts is associated with bilirubin levels exceeding 20 mg/dl in patients experiencing acute liver failure [7]. Our patients exhibited total bilirubin levels below 20 mg/dl; nonetheless, they presented with obstructive jaundice for several months in the first case, and a history of chronic hepatitis C with cirrhosis and possible metastatic lesions in the second case, likely resulting in persistently elevated bilirubin levels and contributing to the development of bile cast nephropathy (BCN).

## Conclusions

The occurrence of acute kidney injury (AKI) is prevalent in patients admitted to the intensive care unit (ICU), leading to a complex hospital course and adverse outcomes. The pathogenesis of AKI in this patient population is multifactorial. While cholemic nephropathy (CN) is a rare etiology of AKI, it is typically linked to elevated bilirubin levels. Nevertheless, instances of CN can also arise in patients with chronic liver disease and lower bilirubin levels.
